# A Case Report of Probable Creutzfeldt-Jakob Disease Based on Positive MRI Findings and the World Health Organization Criteria

**DOI:** 10.7759/cureus.10907

**Published:** 2020-10-12

**Authors:** Syed Asad Ali, Aushna Rasool, Waseem T Malik, Muhammad Tayyab Ilyas

**Affiliations:** 1 Medicine, Shifa International Hospital, Islamabad, PAK; 2 Internal Medicine, Shifa International Hospital, Islamabad, PAK; 3 Neurology, Shifa International Hospital, Islamabad, PAK

**Keywords:** creutzfeldt-jakob disease (cjd), who criteria, mri dwi, cortical ribbon sign

## Abstract

Creutzfeldt-Jakob disease (CJD) is the most common prion disease. It is characterized by neuronal loss, glial cell proliferation, and inflammatory reaction absence. It typically involves deep grey structures, including the caudate nucleus, putamen, and thalamus, with sparing of the hippocampus. Death usually occurs within one year of the onset of symptoms.

A 59-year-old male presented to the outpatient department (OPD) with involuntary jerky movements of his right arm, progressive stiffness of the right half of his body, and slurring of speech for two months. His stiffness had led him to be completely bed-bound. He was admitted and during the hospital stay, he started showing cognitive decline. His MRI brain revealed a bright FLAIR signal in the left basal ganglia, claustrum, sub-, and peri-insular cortex extending into the left parietal parasagittal cortex. He was discharged with a probable diagnosis of CJD with advice to undergo a follow-up MRI brain after one month. He presented again to the hospital after four months with sepsis secondary to urinary tract infection, bedsores, and infected percutaneous endoscopic gastrostomy (PEG) site. His Glasgow Coma Scale (GCS) score on presentation was 8/15, with a fixed gaze and tonic posturing of upper and lower limbs. A follow-up MRI brain showed rapidly progressive cortical atrophy and communicating hydrocephalus consistent with CJD.

The diagnosis of CJD requires the presence of clinical findings with a positive electroencephalogram (EEG), cerebrospinal fluid (CSF) findings, and neuroimaging, or pathological findings. In our patient, a diagnosis of probable CJD was made based on clinical symptoms and positive cortical ribboning on the MRI brain using the World Health Organization (WHO) criteria. EEG was nonspecific, and CSF tau proteins and brain biopsy could not be done.

## Introduction

Creutzfeldt-Jakob disease (CJD) is the most common prion disease [1]; it is a fatal neurodegenerative disorder. It is further subdivided into sporadic (85-95%), familial (5-15%), iatrogenic (less than 1%), and variant forms. The incidence rate of sporadic CJD is one per million per year worldwide, with a mean age of onset between 57-62 years and with no gender predilection. Neuronal loss, glial cell proliferation, and inflammatory response absence are some of the pathological features associated with the condition. Histology shows spongiform changes and the accumulation of abnormal proteins. The process spares the hippocampus and affects deep grey structures, including the caudate nucleus, putamen, and thalamus. Clinical manifestations include rapidly progressive dementia, myoclonus, and pyramidal, extrapyramidal, visual, and cerebellar signs. Death usually occurs within one year of the onset of symptoms.

In this article, we present a case of a probable CJD diagnosed on the basis of patient history, physical findings, and typical MRI brain findings, keeping in view the World Health Organization (WHO) diagnostic criteria for CJD.

## Case presentation

A 59-year-old male, non-smoker, nonaddict, married with six kids, and account officer by profession, initially presented to the neurology outpatient department (OPD). The patient had been in his usual state of health until two months ago when he had started noticing involuntary jerky movements of right arm occurring every two to three minutes. Its frequency had not improved despite treatment with multiple medications. He had also started to experience stiffness in his right upper and lower limb one month ago; initially, he had been up and about, but later, as the stiffness progressed, he had started using support and had gone on to become completely bed-bound. He had started noticing slurring of speech a week before the presentation. He had no history of fever, loss of consciousness, frothing at the mouth, or urinary or fecal incontinence. The rest of the systemic inquiry was unremarkable. Previously, the patient had a history of ischemic heart disease and had undergone percutaneous coronary intervention (PCI) to the left anterior descending artery (LAD) 10 years ago, though his medical treatment had been optimal. His family and social history were unremarkable. The patient had visited multiple hospitals where he had been treated with valproate, haloperidol, and procyclidine. At the time of presentation, he had been taking fluoxetine, propranolol, and carbidopa/levodopa, but his symptoms had been worsening.

The patient was of average height and built, well oriented in time, place, and person with a pulse of 80/min, blood pressure of 130/90 mmHg, respiratory rate of 18/min, and a temperature of 37 °C. Neurological exam showed a Glasgow Coma Scale (GCS) score of 15/15, and an increased tone of the right side of the body with a power of 4/5 in the right upper and right lower limb; reflexes increased on the right side with tremors. The patient also had ataxia. The rest of the examination was unremarkable.

The patient was admitted with suspicion of vascular parkinsonism vs drug-induced extrapyramidal symptoms; however, despite stopping haloperidol, his stiffness did not improve, and he was given a trial of carbidopa/levodopa but continued having myoclonic jerks on the right side of the body, decreased comprehension, disturbed sleep pattern, and agitation. He continued to have cognitive decline and decreased communication with other family members.

**Figure 1:**
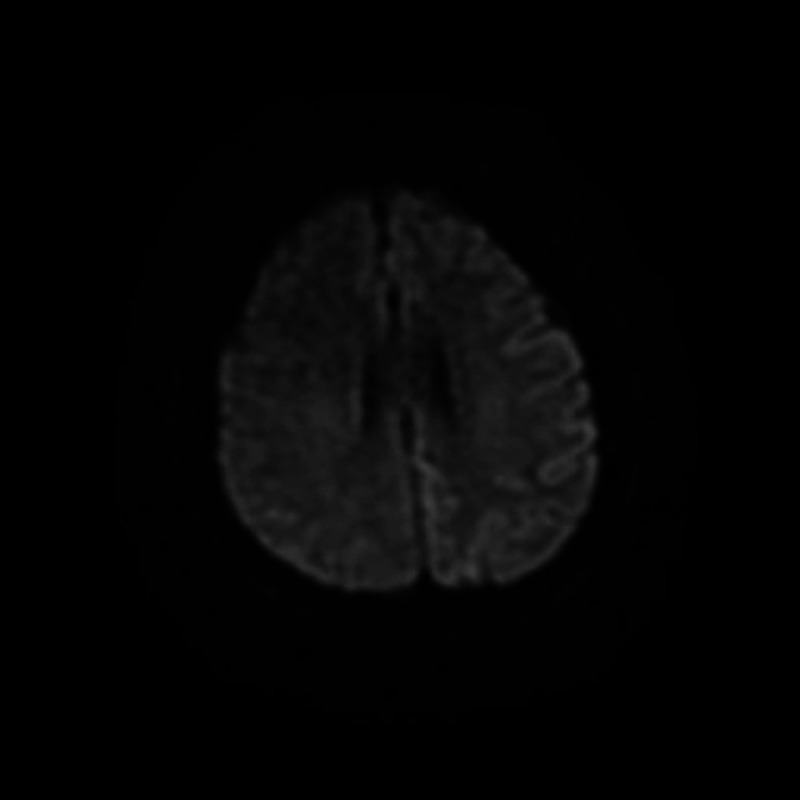
MRI DWI image showing bright signals in various areas of MR cortex consistent with cortical ribboning MRI: magnetic resonance imaging; DWI: diffusion-weighted imaging

**Figure 2:**
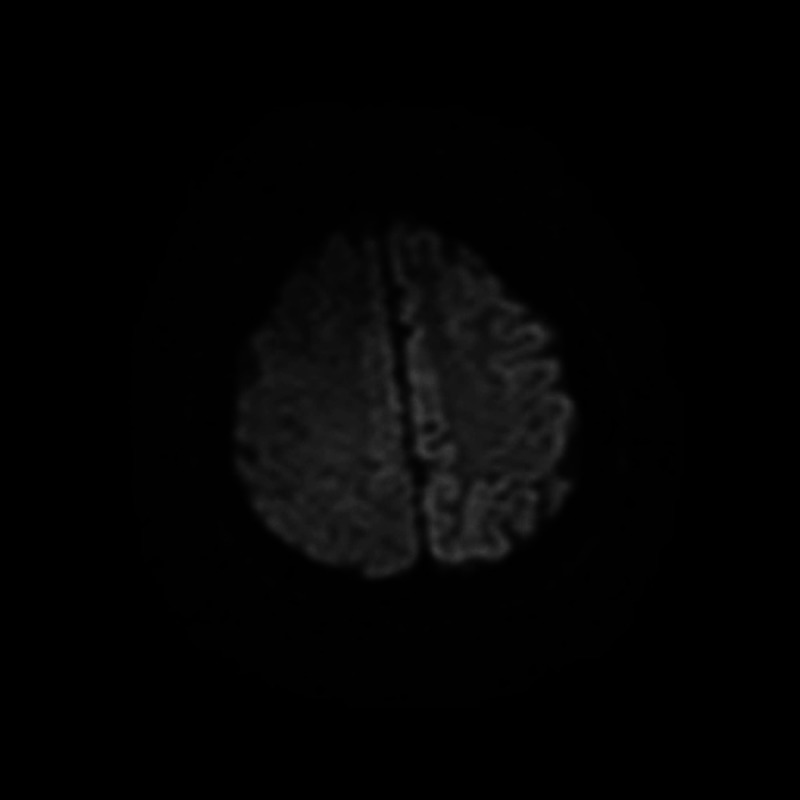
Cortical ribboning The image shows cortical ribboning sign in the left basal ganglia, claustrum, and sub- and peri-insular cortex, and left parietal parasagittal cortex, and the absence of these changes in the corresponding areas on the right side of the brain

His full blood count, serum electrolytes, renal function tests, and liver function tests were normal. Thyroid function tests, parathyroid hormone levels, serum calcium levels, and serum ceruloplasmin levels were also within normal limits. Electroencephalogram (EEG) showed mild diffuse nonspecific encephalopathy with no epileptiform activity. The MRI brain with contrast showed multiple T2, bright FLAIR signal in the periventricular subcortical white matter, consistent with microvascular angiopathy, and subtle bright FLAIR signal in the left basal ganglia, claustrum, and sub- and peri-insular cortex extending into the left parietal parasagittal cortex with normalizing bright diffusion signal concerning for the subacute infarct. CJD was reported as another differential diagnosis (Figures 1, 2).

The autoimmune encephalitis profile and antineuronal profile were also negative. On the basis of rapidly progressive dementia, myoclonus, extrapyramidal signs, and positive MRI brain findings, a diagnosis of probable CJD was made based on the WHO criteria. Cerebrospinal fluid (CSF) analysis for 14-3-3 protein could not be done due to the non-availability of the tests The patient was discharged with an advice to undergo a follow-up MRI after one month and on antipsychotic medications risperidone, clonazepam, piracetam, and valproate.

**Figure 3:**
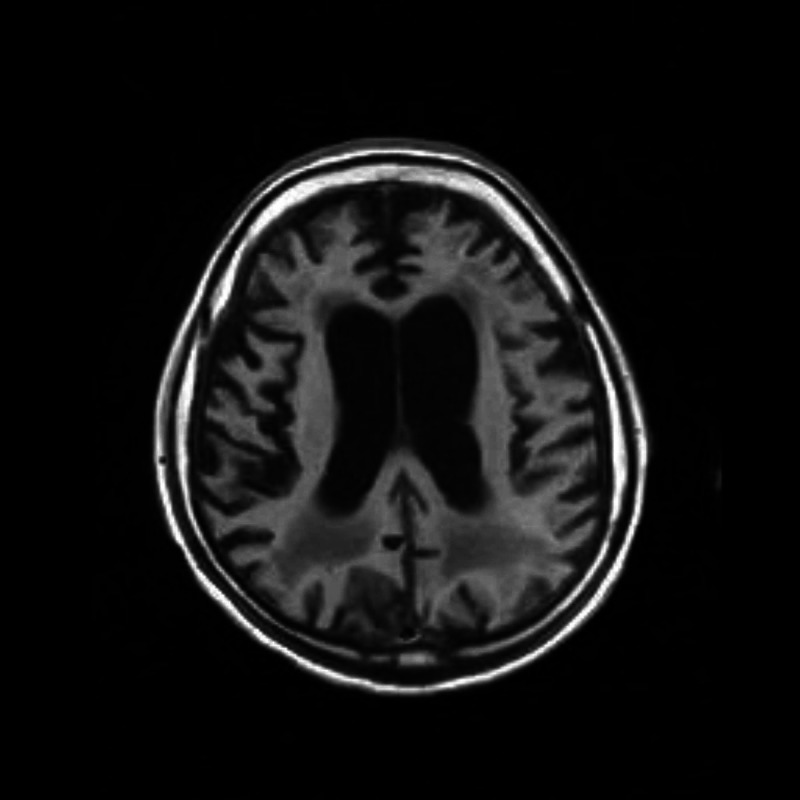
MRI brain showing diffuse cortical atrophy and hydrocephalus MRI: magnetic resonance imaging

The patient presented to the ER after four months with sepsis secondary to bedsores, urosepsis, and infected percutaneous endoscopic gastrostomy (PEG) site. During the past four months, his functional status had declined rapidly. He was completely bed-bound, and his impaired swallowing had led to PEG insertion, and at presentation, his GCS score was 8/15 (E4V1M3). He had a fixed gaze and tonic posturing of limbs with flexed upper and lower limbs. His MRI brain showed an interval increase in previously noted T2 hyperintense signal foci in bilateral periventricular and subcortical white matter, communicating hydrocephalus, and rapidly progressive cortical atrophy consistent with CJD (Figure 3).

## Discussion

The diagnosis of CJD requires the presence of relevant clinical findings along with positive CSF or EEG findings, neuroimaging, or pathological findings.

CSF findings include the presence of 14-3-3 proteins, which has a specificity of 84% and a sensitivity of 94% [2]. EEG findings are variable, ranging from nonspecific diffuse slowing and frontal rhythmic delta activity to periodic sharp wave complexes in the middle and later stages [3].

EEG has a sensitivity of 64% and specificity of 91% in the diagnosis of CJD [4]. MRI findings include increased signal intensity in the putamen, caudate nucleus, and cerebral cortex. A study showed that MRI has a sensitivity of 91% in diagnosing CJD; however, the majority of MRI CJD findings are missed by referral centers [5]. Another study by Shiga et al. showed DW1 MRI to have 92.3% sensitivity and 93.8% specificity [6]. MRI has been found to have much better sensitivity (98%) in diagnosing sporadic CJD compared to CSF proteins [14-3-3: 50%, neuron-specific enolase (NSE): 53%, T-tau: 68%] [7].

Biopsy or autopsy findings show neuronal loss, gliosis, and spongiform changes, which aid in definitive diagnosis. Abnormal prion proteins PrPsc detection and molecular classification have diagnostic value [8]. However, the practice of conducting post-mortem examinations of patients with suspected CJD is scarce for multiple reasons [9].

The WHO has established definitive criteria for diagnosing CJD, taking into account different diagnostic modalities and variances in clinical presentations [10]. Considering these criteria, our patient had rapidly progressive dementia along with myoclonus, nystagmus, and increased rigidity of his right side. MRI showed increased signals in the caudate nucleus along with cortical ribboning, which makes CJD a probable diagnosis. EEG findings were nonspecific in our patient, and the CSF protein 14-3-3 testing was not available in our set-up. Post-mortem examination of the patient could not be done due to the family's refusal to give consent.

## Conclusions

We presented a case of sporadic CJD and discussed the use of proper diagnostic approaches and tools. DWI MRI, being non-invasive and having a better sensitivity, should be used to diagnose CJD in settings where the options of CSF protein test and biopsy are not available.
